# Chemotherapeutic errors in hospitalised cancer patients: attributable damage and extra costs

**DOI:** 10.1186/1471-2407-11-478

**Published:** 2011-11-08

**Authors:** Florence Ranchon, Gilles Salles, Hans-Martin Späth, Vérane Schwiertz, Nicolas Vantard, Stéphanie Parat, Florence Broussais, Benoît You, Sophie Tartas, Pierre Jean Souquet, Claude Dussart, Claire Falandry, Emilie Henin, Gilles Freyer, Catherine Rioufol

**Affiliations:** 1 Hospices Civils de Lyon, Groupement Hospitalier Sud, Clinical Oncology Pharmacy Department, Pierre Bénite - Université Lyon 1, Ecole Doctorale Interdisciplinaire Sciences Santé, Lyon, France; 2 Hospices Civils de Lyon, Groupement Hospitalier Sud, Department of Haematology, Pierre Bénite. Université Lyon 1, UMR5239 CNRS -ENS, Lyon, France; 3 Université Lyon 1, EA 4129 « Santé Individu et Société », Lyon, France; 4 Hospices Civils de Lyon, Groupement Hospitalier Sud, Clinical Oncology Pharmacy Department, Pierre Bénite, France; 5 Hospices Civils de Lyon, Groupement Hospitalier Sud, Department of Haematology, Pierre Bénite, France; 6 Hospices Civils de Lyon, Groupement Hospitalier Sud, Department of Oncology, Pierre Bénite- Université Lyon 1, EMR UCBL/HCL 3738, Lyon, France; 7 Hospices Civils de Lyon, Groupement Hospitalier Sud, Department of Oncology, Pierre Bénite, France; 8 Hospices Civils de Lyon, Groupement Hospitalier Sud, Department of Pneumology, Pierre Bénite, France; 9 Hôpital d'Instructions des Armées Desgenettes, Department of pharmacy, Lyon - Université Lyon 1, Ecole Doctorale Interdisciplinaire Sciences Santé, Lyon, France; 10 Université Lyon 1, EMR UCBL/HCL 3738, France; 11 Hospices Civils de Lyon, Groupement Hospitalier Sud, Clinical Oncology Pharmacy Department, Pierre Bénite - Université Lyon 1, EMR UCBL/HCL 3738, Lyon, France

## Abstract

**Background:**

In spite of increasing efforts to enhance patient safety, medication errors in hospitalised patients are still relatively common, but with potentially severe consequences. This study aimed to assess antineoplastic medication errors in both affected patients and intercepted cases in terms of frequency, severity for patients, and costs.

**Methods:**

A 1-year prospective study was conducted in order to identify the medication errors that occurred during chemotherapy treatment of cancer patients at a French university hospital. The severity and potential consequences of intercepted errors were independently assessed by two physicians. A cost analysis was performed using a simulation of potential hospital stays, with estimations based on the costs of diagnosis-related groups.

**Results:**

Among the 6, 607 antineoplastic prescriptions, 341 (5.2%) contained at least one error, corresponding to a total of 449 medication errors. However, most errors (n = 436) were intercepted before medication was administered to the patients. Prescription errors represented 91% of errors, followed by pharmaceutical (8%) and administration errors (1%). According to an independent estimation, 13.4% of avoided errors would have resulted in temporary injury and 2.6% in permanent damage, while 2.6% would have compromised the vital prognosis of the patient, with four to eight deaths thus being avoided. Overall, 13 medication errors reached the patient without causing damage, although two patients required enhanced monitoring. If the intercepted errors had not been discovered, they would have resulted in 216 additional days of hospitalisation and cost an estimated annual total of 92, 907€, comprising 69, 248€ (74%) in hospital stays and 23, 658€ (26%) in additional drugs.

**Conclusion:**

Our findings point to the very small number of chemotherapy errors that actually reach patients, although problems in the chemotherapy ordering process are frequent, with the potential for being dangerous and costly.

## Background

The report, "To Err is Human", from the Institute of Medicine estimated that between 44, 000 and 98, 000 patients die each year in the USA as a result of medical errors [1]. Although certain adverse events are unavoidable, many are preventable, with medication errors being a major cause of such accidents [2]. Medication errors may occur anytime and at any stage during the medication use process, from the prescription of the drug to its preparation, dispensing, and final administration to the patient. Moreover, the medication process involves the whole medical team, involving physicians, pharmacists, and nurses [3].

Medication errors with antineoplastic drugs may be catastrophic due to the drugs' high toxicity and small therapeutic index in addition to the health status of cancer patients. A study revealed that antineoplastic agents were the second most common cause of fatal medication errors [4]. While overdosage is likely to result in permanent damage to the patient, underdosage may compromise the success of therapy. Although several individual case reports focused on medication errors [5-8], some of which were fatal [9-12], an overview of the issue is still needed.

At present, the prevention of antineoplastic medication errors is a priority in hospitals [13], with numerous recommendations being published in order to help decrease the likelihood of errors [14-16]. The emerging patient safety movement advocated a shift from the historical culture of blame and shame to a culture of transparency in order to encourage health care workers to report their errors [17]. Consequently, national reporting systems for medication errors were established, but as these were not mandatory, the published errors tended to reflect only a small proportion of the actual errors committed. Little data is available on the potential severity and clinical consequences of antineoplastic medication errors in terms of the need for enhanced patient monitoring, new or prolonged hospitalisation, and initiation of new treatments. Medication errors are costly to patients, their families, and employers, as well as to hospitals, health-care providers, and insurance companies, although there are few reliable estimates of their costs [18,19].

In order to reduce medication errors, we must increase our awareness about their occurrence and consequences. Thus, we conducted a 1-year prospective study aimed at detecting antineoplastic medication errors in both affected patients and intercepted cases in terms of frequency, severity for patients, and economic impact on the French Public Health Insurance.

## Methods

### Setting

The study was conducted in a 1200-bed teaching hospital in France. During the 1-year study, the centralised cytotoxic preparation unit set up approximately 21, 000 doses of antineoplastic agents for patients in the haematology, medical oncology, and pneumology wards, as well as other units involved in the care of cancer patients. Wards included both daycare and inpatient units. The age, experience, and status of prescribers varied, ranging from residents to senior attending physicians. The medical data system was not fully computerised as prescriptions were handwritten on standardised forms, entered into a local database, and then transferred to the pharmaceutical unit in order to be verified by pharmacists. In addition, all forms were validated by senior physicians and pharmacists. Physicians had to approve the prescription, providing a signed "green light" for both cytotoxic drug preparation and administration, indicating that they thought patients were able to receive the treatment according to their daily clinical and biological status (blood count, *etc*.). Pharmacists had to analyse prescriptions by verifying patient data (identity, age, weight, and height) as well as the antineoplastic regimen to be used, and then entering the data into dedicated pharmacy software (Asclepios^®^). In addition, pharmacists confirmed any dose adjustment or deviation from the validated antineoplastic regimen. The software calculated other data, namely body surface area, anticancer drug dose, date and time of prescription, as well as day and duration of drug delivery to the patient. Fabrication forms that permitted the preparation of anticancer drugs by pharmacy technicians were also edited, including a double check at each step of the fabrication process. Prior to dispensing prescriptions to medical wards, for each preparation, pharmacists carried out a qualitative control by verifying the patient's name, drug dose, and type and volume of dilution fluid in addition to a semi-quantitative control by comparing the ordered dose and number of vials used.

### Detection of medication errors

Our prospective study undertaken between June 2006 and May 2007 aimed to collect all antineoplastic medication errors, concerning both unintercepted mistakes that affected patients and intercepted mistakes. Medication errors were defined as a failure in the treatment process, which led to or had the potential to lead to the patient being harmed. The different types of medication errors are defined in Table 1. During routine practice, errors were able to be detected and intercepted at every step of the chemotherapy process, with all health professionals being involved in error detection. Prescription errors were detected by pharmacists using systematic pharmaceutical analysis of all prescribed antineoplastic regimens. Preparation errors were detected during the preparation by the double checked of the fabrication process and by self-reported by pharmacy technicians, or at the time of final pharmaceutical control. Finally, administration errors were reported on a voluntary basis by nurses or physicians.

**Table 1:** Classification of medication errors

Type of medication errors	Definition
Prescription errors	
Error linked to the choice of antineoplastic regimen	Difference of antineoplastic regimen compared to the previous cycle or the multidisciplinary medical decision
Dose error	Under- or overdosage of more than 5% of antineoplastic drugs (calculation mistake or omission of dose reduction when dosage adjustment was required)
Incomplete prescription	Missing data on the prescription, such as patient identity, anthropometric or biological data, drug dose, prescriber's signature, and date of administration
Cancellation of medical approval	Misinterpretation of the clinical status of the patient, who was not able to receive chemotherapy
Pharmaceutical errors	Errors in pharmaceutical analysis (*i.e.*, pharmacist-generated mistakes or failure to detect prescription errors), data entry in the pharmaceutical software, preparation, storage, or dispensing errors
Drug administration errors	Any discrepancy between the physicians' chemotherapy orders and drug delivery to the patient, such as timing errors, omission, unordered drug, wrong route, wrong patient, deteriorated drug, and technical error in administration (*e.g*., wrong infusion flow rate)

### Analysis of potential clinical consequences

For each intercepted medication error, the potential severity was evaluated according to the Medication Error Index for categorising such errors, ranging in severity from "no patient harm" to "potential patient death" [3]. A literature review on chemotherapy medication errors and their consequences was performed using Pubmed database, with the search keywords being "antineoplastic agents and overdoses" and "antineoplastic agents and medication errors". An analysis of case reports allowed us to design a worksheet aimed to assess the potential clinical consequences arising from anticancer medication errors in terms of the need for enhanced patient monitoring, hospitalisation (number and duration of hospital stays), or initiation of new treatments (Table 2). The potential severity and consequences of the avoided errors were assessed and scored independently by two physicians specialised in haematology, oncology, and pneumology according to the case. Based on our analysis of actual prescription charts and error descriptions, the assessment of the potential damage of each error took into account individual patient history and pharmacological data regarding the risk of adverse events. Two pharmacists analysed the remaining intercepted medication errors that did not have any impact on patients.

**Table 2:** Worksheet used to assess the potential clinical consequences due to anticancer medication errors

Error number: Check the appropriate boxes	Physician assessment number: 1 or 2	
		
Potential severity according to the Medication Error Index (3)		
		
No consequences for the patient		
		
An error avoided that would not cause patient harm		
An error avoided that would require monitoring to confirm that it did not result in patient harm		
**Temporary damage**	**Potential clinical consequences**	Potential hospitalisation and treatment*

An error avoided that could have contributed to temporary harm to the patient, requiring intervention	Acute renal failure	**Hospitalisation**
	Cardiac toxicity	Prolongation of the initial hospitalisation
	Skin toxicity	New hospitalisation
An error avoided that could have contributed to temporary harm to the patient, requiring initial or prolonged hospitalisation	Neutropenia	**Type of ward**
	Thrombopenia	Standard hospitalisation unit
	Anemia	Day-care unit
	Febrile neutropenia	Intensive care unit
	Neurotoxicity	**Number of additional days of hospitalisation**
		
**Permanent damage**	Hepatic cytolysis	**Treatments**
		
An error avoided that could have contributed to permanent patient harm	Hepatic cholestasis	Platelet transfusions
		
**Compromised vital prognosis**	Diarrhea	Erythrocyte transfusions
		
An error avoided that could have required intervention necessary to sustain life	Vomiting	Granulocyte Colony Stimulating Factor
		
**Patient death**	Other:	Parenteral nutrition
		Other:
An error avoided that could have contributed to the patient's death		

* Only drugs funded in addition to the diagnosis-related groups costs

### Cost simulations

Cost was defined as the hospital costs incurred by the French public health insurance system in order to treat the victims of antineoplastic medication errors. In theory, these resources would not have been required had there been no error. Three scenarios were thus possible:

1- The chemotherapy medication error would have been without clinical consequences for the patient, and without economic consequences for the hospital.

2- The chemotherapy medication error would have resulted in clinical consequences for the patient, requiring out-of-hospital treatment, but without economic consequences for the hospital (for example, a minor overdosage of vincristin causing peripheral neurotoxicities, which might be treated at home using analgesics).

3- The chemotherapy medication error would have resulted in clinical consequences for the patient, requiring hospitalisation and treatment. In this case, hospital stays depending on potential consequences of medication errors as estimated by physicians were simulated using GHIM software (Hospital and medical information management) based on the diagnosis-related groups of the French medical hospital information system. These simulations were conducted according to established guidelines [20]. The costs of expensive drugs, which were funded by the French public health insurance system, in addition to costs from diagnosis-related groups were taken into account [21].

### Statistical analysis

For the descriptive analysis of medication errors, the unit of analysis was the number of errors, with a prescribing medication order containing one or more drugs considered to correspond to one or more medication errors. The unit of analysis for assessing severity and costs was the prescribing medication order.

Univariate analysis was conducted using the Chi-squared test in order to compare the number of medication errors in relation to the month of the year or medical ward. The reliability of physicians' judgments was calculated using Kappa statistics [22], with all statistical analyses being performed using SPSS^®^.

## Results

### Chemotherapy medication errors: incidence and type

During the 12-month study period, the pharmacy unit received 6, 607 prescriptions corresponding to 22, 138 distinct anticancer drugs. In total, 449 medication errors were detected throughout the medication use process, involving 341 prescriptions (Table 3). The overall medication error rate was 5.2%. Among the 449 medication errors, 436 were intercepted by physicians, pharmacists, or nurses prior to administration, while 13 reached the patients (2.9% of all errors). Medication errors were made by residents (50.7%), residents with approval by a senior oncologist (19%), and senior physicians (30.3%).

**Table 3:** Descriptive analysis of frequency and type of chemotherapy medication errors over 1 year

	Number of errors					
**Type of chemotherapy medication errors**	**Haematology**	**Medical Oncology**	**Pneumology**	**Others specialities**^**1**^	**Pharmacy**	**Total**

**Prescription error**						
Erroneous choice of antineoplastic regimen	20	5	3	3	-	31
Data missing on the prescription	128	28	25	15	-	196
Wrong dose						
< 10%	13	11	11	4	-	39
[10-50%[	26	18	28	15	-	87
[50-100%]	15	9	2	4	-	30
> 100%	2	4	3	2	-	11
Withdrawal of medical approval	5	3	5	1	-	14

*Total (1)*	*209*	*78*	*77*	*44*	*-*	*408*

**Pharmaceutical error**						
Pharmaceutical analysis	-	-	-	-	4	4
Data entry	-	-	-	-	4	4
Fabrication	-	-	-	-	26	26
Storage	-	-	-	-	1	1
Dispensation	-	-	-	-	1	1

*Total (2)*	*-*	*-*	*-*	*-*	*36*	*36*

**Drug administration error**						

*Total (3)*	*3*	*-*	*-*	*2*	*-*	*5*

Total (1+2+3)	212	78	77	46	36	449

**Number of antineoplastic agents prepared by pharmacy**	11866	5270	2743	2259	22138	-

**Percentage of medication errors**	1.79	1.48	2.81	2.04	0.16	-

^1^Others specialities included gastroenterology (23 medication errors) and radiotherapy (23 medication errors)

Approximately 91% (408/449 errors) of medication errors concerned inadequate prescriptions, with 405 being intercepted. Overall, 31 errors were linked to the choice of antineoplastic regimen (7.6%). In 196 cases, prescriptions were incomplete with data missing from the prescription (48%), while in 167 cases, erroneous medication doses were recorded (40.9%). The most common causal drug was carboplatin, which was involved in 35 cases or 21% of dose errors, despite corresponding to only 3% of anticancer drugs prescribed at our institution. The most prescribed drug, 5-fluorouracil, was involved in 12% of all prescriptions and represented 11% of dose errors (19 cases), while oxaliplatin represented only 2% of prescriptions, but was the third most common drug involved in dose errors (8% or 13 cases) (Table 4). In 14 cases (3.4%), the physician requested the drug preparation process to be stopped in order to further analyse the patient's status despite previously giving medical approval.

**Table 4:** Descriptive analysis of antineoplastic dose errors

	Underdosage				Overdosage					
**Percentage of error**^**1**^	**< 10**	**[10-50[**	**[50-100]**	**> 100**	**< 10**	**[10-50[**	**[50-100]**	**> 100**	**Total**	**Frequency of drug prescriptions (%)**

Bevacizumab	-	-	2	-	1	-	-	-	3	0.65
Bleomycin	-	-	-	-	-	3	-	-	3	3.1
Bortezomib	1	-	-	-	-	3	1	-	5	3.36
Carboplatin	4	9	1	-	5	14	2	-	35	2.66
Cetuximab	-	-	-	-	-	-	2	-	2	0.99
Cisplatin	-	-	-	-	-	2	1	3	6	5.22
Cyclophosphamide	1	2	1	-	1	2	1	-	8	6.31
Cytarabine	-	1	-	-	-	-	-	-	1	3.23
Dacarbazine	-	-	-	-	-	1	-	-	1	1.71
Docetaxel	-	-	-	-	-	2	-	1	3	2.69
Doxorubicin	-	-	1	-	2	-	-	1	4	8.04
Drug in clinical trial	-	-	1	-	-	1	-	-	2	0.5
Liposomal doxorubicin	-	-	-	-	-	2	-	-	2	0.31
Epirubicin	-	-	1	-	-	-	-	-	1	0.6
Etoposide	1	1	-	-	1	1	-	1	5	7.82
Fluorouracil	4	-	1	-	4	9	1	-	19	12.1
Gemcitabine	-	1	-	-	-	5	3	1	10	4.91
Ifosfamide	-	2	2	-	2	2	-	-	8	2.67
Irinotecan	1	-	-	-	1	1	-	1	4	2.57
Methotrexate	-	-	1	-	-	1	-	-	2	1.6
Oxaliplatin	1	2	-	-	1	7	1	1	13	2.15
Paclitaxel	1	-	-	-	-	3	-	-	4	1.63
Raltitrexed	-	-	-	-	-	-	1	-	1	0.1
Rituximab	-	2	-	-	4	-	1	-	7	8.21
Trastuzumab	-	1	1	-	-	-	-	-	2	1.78
Vinblastine	-	-	-	-	1	1	-	1	3	2.01
Vincristine	-	-	-	-	1	4	3	-	8	5.18
Vindesine	-	-	-	-	1	-	-	-	1	0.75
Vinorelbine	-	-	-	-	-	2	1	1	4	2.12

Total	14	21	12	-	25	66	18	11	167	

^1 ^Percentage of error = (theoretical dose - incorrect dose)/theoretical dose *100

Overall, 36 pharmaceutical errors occurred, involving 0.16% (36/22, 138) of all anticancer drugs prepared, with 30 errors being intercepted. Pharmaceutical errors were classified as resulting from pharmaceutical analysis (4), data entry into the pharmaceutical software (4), preparation (26), storage (1), or dispensing errors (1).

Five errors of drug administration were reported by nurses or physicians, or 0.02% (5/22, 138) of all anticancer drugs given to patients, with only one error being intercepted just prior to administration.

**Figure 1:**
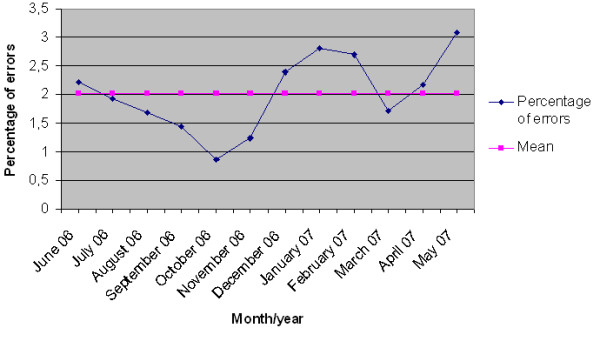
**Percentages of medication errors by month of the year**.

A statistically significant relationship was found between the rate of medication errors and month of the year (p = 0.001). May and January were the months most at risk of errors, while October and November were the least (Figure 1). When taking into account medical wards, error rates were significantly lower in haematology and medical oncology (p = 0.001, Table 3).

### Medication errors: severity

In total, 341 erroneous prescriptions were reported, with 329 being intercepted. For 191 of the erroneous prescriptions considered to be without impact, the potential severity was assessed by the pharmaceutical team. The remaining 138 cases of intercepted prescriptions (41.9% of erroneous protocols) were analysed by two independent physicians from the haematology, oncology including gastroenterology and radiotherapy, and pneumology wards (Table 5). The concordance of medical judgement was found to vary depending on the medical speciality, being good in haematology (k = 0.75, 53 prescriptions), but moderate in oncology (k = 0.51, 48 prescriptions) and pneumology (k = 0.42, 37 prescriptions). Overall, 81.4% of intercepted medication errors would have had no impact for the patients. However, 13.4% of errors would have resulted in temporary damage and 2.6% in permanent injury, while 2.6% would have compromised the vital prognosis of the patient. The potential injuries from medication errors would have varied, with 40 cases of haematological toxicity, 27 of neurotoxicity, six of hepatic cytolysis, nine of renal failure, three of skin toxicity, and two of cardiac toxicity being avoided. If not intercepted, between four and eight medication errors would have resulted in the patient's death. These avoided incidents related to eight overdosages and one wrong route of administration. The drugs involved in the averted fatal overdosages were vinblastine (592.5 mg prescribed instead of 9.48 mg), vinorelbine (300 mg instead of 30 mg), cisplatin (1, 344 mg instead of 134 mg, a daily dose of 92 mg instead of 34 mg for 5 successive days), doxorubicin (415 mg instead of 41.5 mg), and docetaxel (918 mg instead of 85.5 mg). In addition, two cases of ten-fold dose errors were intercepted with the prescription of 1, 830 mg of etoposide instead of 183 mg and 1, 830 mg of cisplatin instead of 183 mg. The wrong administration route error concerned the erroneous intrathecal administration of intravenous vincristine, which was intercepted just in time in the medical ward.

**Table 5:** Severity distribution of intercepted medication errors

	Number of protocols with at least one error							
**Potential severity according to the Medication Error Index (3)**	**Haematology**		**Oncology **^**1**^		**Pneumology**		**Pharmacy**	**Total^2 ^(% of erroneous prescription)**
	**a**	**b**	**c**	**d**	**e**	**f**	**g**	

**No consequences for the patient**								
An error avoided that would not cause patient harm	20	21	18	19	14	14	157	210
An error avoided that would require monitoring to confirm that it did not result in patient harm	3	10	4	8	7	16	34	58
*Total*								*268 (81.4%)*
**Temporary damage**								
An error avoided that could have contributed to temporary harm to the patient, requiring intervention	6	0	9	8	9	4	0	18
An error avoided that could have contributed to temporary harm to the patient, requiring initial or prolonged hospitalisation	14	12	13	6	6	1	0	26
*Total*								*44 (13.4%)*
**Permanent damage**								
An error avoided that could have contributed to permanent patient harm	7	7	1	2	0	0	0	*8.5 (2.6%)*
**Compromised vital prognosis**								
An error avoided that could have required intervention necessary to sustain life	0	0	2	2	1	0	0	*2.5 (0.8%)*
**Patient death**								
An error avoided that could have contributed to the patient's death	3	3	1	3	0	2	0	*6 (1.8%)*
**Total**	53	53	48	48	37	37	191	329 *(100%)*

^1 ^including gastroenterology and radiotherapy

^2 ^Total = (a+b)/2 + (c+d)/2 + (e+f)/2 +g

A total of 13 medication errors reached patients. One of these errors required enhanced monitoring of a diabetic patient, after administering anticancer preparations with a glucose solvent. Another error impacted anticoagulant treatment without any injury to the patient. The remaining errors had no consequences for the patients involved.

### Cost evaluation

During the 12-month period, the potential cost of the intercepted medication errors to the French health insurance system was estimated at 92, 907€, with 69, 248€ (74%) attributed to hospitalisation and 23, 658€ (26%) to the cost of drugs in addition to the diagnosis-related groups (Table 6). Furthermore, if not intercepted, the medication errors described above would have led to 216 additional hospital days. The results from the evaluation by two physicians according to speciality are provided in Table 6, reflecting the divergence in physicians' medical appreciation of medication errors and their clinical consequences.

**Table 6:** Assessment of the potential cost of medication errors

	Haematology		**Oncology**^**1**^		Pneumology		
	a	b	c	d	e	f	**Total**^**2**^
Cost linked to new potential hospitalisation (€ 2008)	7, 908.5	5, 354.2	3, 614.3	844.9	0	0	8, 861
Cost linked to potential prolongation of hospitalisation (€ 2008)	26, 720.9	29, 213.8	24, 039.5	21, 232.3	13, 259.6	6, 309.1	60, 387.6
Cost linked to drugs paid for in addition to the diagnosis-related groups (€ 2008)	8, 313.9	8, 383.9	12, 378.8	12, 378.8	2, 930.9	2, 930.9	23, 658.6

Total (€ 2008)	42, 943.3	42, 951.9	40, 032.6	34, 456	16, 190.5	9, 240	92, 907.2

^1 ^including gastroenterology and radiotherapy

^2 ^Total = (a+b)/2 + (c+d)/2 + (e+f)/2

## Discussion

Our results demonstrate that medication errors occurred frequently at our hospital, at a rate of 5.2%, which is comparable to the rates reported in published literature, ranging from 0.4% to 31.9% [23-25]. The significant relationship found between the rate of medication errors and month of the year is still unclear, as it cannot be accounted by the biannual rotation of residents occurring in May and November. Furthermore, error rates were lower in the haematology and medical oncology wards (p = 0.001), where the most antineoplastic chemotherapy was prescribed, suggesting that oncological experience was incremental in reducing the error risk. Indeed, the haematology and medical oncology wards are specialised in cancer treatment, while the pneumology and gastroenterology wards usually treat other diseases. In addition, in the haematology and medical oncology wards, residents and physicians generally attend a specific training program, including more detailed practical and theoretical approaches dedicated to cancer chemotherapy treatments. Incomplete prescriptions were found to be frequent and time-consuming for physicians and pharmacists, constituting a delaying factor in the setting up of therapy. Medication dose errors represented the second most common type of error in terms of frequency, with the dose difference being > 50% higher than the theoretical dose in 24.6% of cases (n = 41). These dose errors are considered to be the most dangerous for the patient. In our study, very few prescription medication errors actually reached the patient, due to the dedicated quality control system. Most prescription errors could possibly have been avoided by using a computerised chemotherapy prescribing system [26], but such a system is not available in all French hospitals. However, more recent publications highlighted the role played by computerised systems in the occurrence of other types of errors, demonstrating computers to be only a part of the ongoing process aimed to improve patient safety [27-29].

Only five administration errors (0.02%) were detected, which was lower than the rate reported in literature [30,31]. The pharmaceutical error rate (0.16%) appeared to be very low compared with a similar study focused on the preparation process (3.6% of pharmaceutical errors) [32] and also lower than another study using a totally computerised chemotherapy prescribing system (0.45%) [33]. Bibliographic data suggests that there was significant under-reporting of administration and pharmaceutical errors in our study, which may be explained by a number of factors, such as the fear of blame as well as the bureaucratic and time-consuming process for reporting incidents.

Our findings provide more precise information than previously available on the potential severity and clinical consequences of medication errors in terms of required monitoring, hospitalisation, and treatment. In our study, if medication errors had not been intercepted prior to administration, 13.4% would have caused temporary damage and 2.6% permanent injury, while 2.6% would have compromised the vital prognosis of the patient. The potential for fatal accidents still exists, with four to eight deaths being avoided by means of the collaborative medical and pharmaceutical teams. The dramatic consequences of antineoplastic medication errors are crucial given the nature of anticancer drug toxicity, the use of antineoplastics drugs in complex multiple-drug regimens, and the overall health status of cancer patients. In our study, the potential consequences of medication errors could not be established, but instead they were estimated by physicians, which was a difficult exercise as illustrated by the kappa score. Indeed, some toxicities, such as neurotoxicities, along with their impact on patient care are difficult to predict. Furthermore, the evaluations were arbitrary, as they did not take into account the measures adopted in case of immediate discovery of the medication errors.

To our knowledge, no studies to date have estimated the potential costs of antineoplastic medication errors. It was estimated that close to 100, 000€ was saved in addition to 216 additional days of hospitalisation. Comparisons with other cost analysis studies are difficult, due to the differing methodologies, choices, and specific features of the health systems in various countries. In addition, our study was limited to antineoplastic chemotherapy, which in turn reduces its comparability, and our cost analysis took only into account hospital and medication costs arising for the French health insurance system. However, the cost of medication errors for the society as a whole is much higher, when considering the direct non-medical costs, such as patient transportation, home care, and housing fitting if necessary, and the indirect costs, like hiring home help, as well as the intangible costs, such as those relating to pain and suffering. In addition, it should be noted that the costs involved in the time spent by physicians, pharmacists, and nurses on the quality control system aimed at intercepting medication errors was not evaluated in our study, although an economic assessment of this issue appears warranted.

## Conclusion

This study demonstrated that very few medication errors actually reached patients, although defects in the chemotherapy ordering process were frequent, with the potential to be dangerous and costly. Oncology was an area of particular risk due to the severity of medical consequences for the patient, as shown by the six potential deaths that were avoided. The potential costs related to medication errors were not negligible, being nearly 100, 000€ over a 1-year time period, although this figure did not include indirect outpatient medical and other non-medical related costs. The dedicated quality control system was able to intercept most of the medication errors prior to administration. Such a chemotherapy control system, however, requires close cooperation between physicians, pharmacists, and nurses.

Our study highlighted the need for developing systematic preventive actions in order to reduce medication errors and improve quality of care. Electronic prescribing of antineoplastic chemotherapy appears to be the next step in the ongoing process of improving safety. Other complementary approaches, such as medication error reviews that allow for a collective and multidisciplinary error analysis, should also be implemented.

## Competing interests

The authors declare that they have no competing interests.

No direct funding was received for this study. The authors were personally salaried by their institutions during the period of writing, although no specific salary was set aside or given for writing the paper.

## Authors' contributions

All authors participated in the study, read the manuscript, and provided their final approval.

## Acknowledgements and funding

We would like to acknowledge the medical, pharmaceutical, and nursing teams at the *Centre Hospitalier Lyon Sud, Hospices Civils de Lyon*, France.

No direct funding was received for the study. The authors were personally salaried by their institutions during the period of writing, although no specific salary was set aside or given for writing the paper.

## Pre-publication history

The pre-publication history for this paper can be accessed here:

http://www.biomedcentral.com/1471-2407/11/478/prepub
